# Qizhi Jiangtang Jiaonang Improves Insulin Signaling and Reduces Inflammatory Cytokine Secretion and Reactive Oxygen Species Formation in Insulin Resistant HepG2 Cells

**DOI:** 10.1155/2015/518639

**Published:** 2015-05-05

**Authors:** Xiao-Tian Zhang, Chun-Jiang Yu, Jian-Wei Liu, Yan-Ping Zhang, Chao Zhang, Chen-Xue Song, Jing-Shu Xie, Jing-Ying Sai, Jing-Tong Zheng, Fang Wang

**Affiliations:** ^1^Department of Pathogenobiology, The Key Laboratory of Zoonosis, Chinese Ministry of Education, College of Basic Medicine, Jilin University, Changchun, Jilin 130021, China; ^2^Jilin Yizheng Pharmaceutical Group Co., Ltd., Siping, Jilin 136001, China; ^3^The Second Department of Cardiology, The people's Liberation Army No. 208 Hospital, Changchun, Jilin 130021, China

## Abstract

We analyzed the effects of a traditional Chinese medicine, Qizhi Jiangtang Jiaonang (QJJ), on insulin resistance (IR) in vitro. After an in vitro model of IR was established by treating human liver cancer cells (HepG2 cells) with palmitic acid, the cells were then treated with various concentrations of QJJ. Treatment with 400 *µ*M palmitic acid for 24 h induced IR in HepG2 cells. The survival rate for HepG2 cells in the IR group was significantly lower than that of the untreated control group (*P* < 0.001); however, QJJ restored HepG2 cell survival (*P* < 0.001). As compared with HepG2 cells in the IR group, QJJ at all doses analyzed significantly increased glucose consumption (all *P* < 0.05). Moreover, treatment with all the QJJ doses significantly reduced the mean intracellular reactive oxygen species levels as compared with the IR group (all *P* < 0.05). Furthermore, high-dose QJJ reduced both TNF-*α* and IL-6 levels as compared to the IR group (all *P* < 0.05). QJJ ameliorated the altered PI3K, GLUT4, and RAGE expression observed with IR. In conclusion, QJJ can improve IR in HepG2 cells, which may be mediated through the IRS-1/PI3K/GLUT4 signaling pathway as well as regulation of NF-*κ*B-mediated inflammation and oxidative stress.

## 1. Introduction

The incidence of type 2 diabetes mellitus (T2DM) has dramatically increased in both developed and developing countries; the World Health Organization (WHO) projects it to become the 7th leading cause of death worldwide [1]. Although the exact causes of T2DM are still under debate, accumulation of ectopic fat via lipotoxicity is thought to lead to the insulin resistance (IR) and hyperglycemia that underlie the pathogenesis of T2DM [2, 3]. IR refers to the reduced biological response to insulin or the relative insufficiency of insulin and is characterized by reduced sensitivity to insulin and disordered use of glucose in peripheral tissues. Improper management of IR can result in a range of complications, including heart disease, stroke, diabetic retinopathy, kidney failure, and loss of limbs.

The effects of insulin are mediated through the autophosphorylation of its receptor, insulin receptor (InsR), a transmembrane protein composed of *α* and *β* subunits. Activated InsR subsequently phosphorylates tyrosine residues within insulin receptor substrate (IRS) proteins, which then activates phosphatidylinositol 3-kinase (PI3K) [4]. This induces protein kinase B (PKB/Akt) translocation to the cell membrane where it is activated [5], thereby inducing gene expression and promoting the translocation of glucose transporter 4 (GLUT4) into the cell membrane of liver, adipose, and skeletal muscle cells [6], facilitating glucose uptake, and improving plasma glucose levels. Impaired binding of insulin to its receptor or suppression of the insulin signal transduction may cause IR [7]. Inhibition of IL-6 signaling by MR16-1 increased skeletal muscle GLUT4 and PPAR*γ* expression, reducing plasma glucose levels independent of insulin stimulation, and prevented hepatic steatosis in mice fed a high-fat diet [8].

Aerobic exercise [9] and consumption of a low glycemic index diet [10] are known to improve insulin sensitivity. In addition to lifestyle modification, metformin, which inhibits hepatic glucose production, may also improve glycemic control in individuals with T2DM; however, it is recommended for only those with normal kidney function. The benefits of combination regimens of metformin plus glucagon-like peptide-1 (GLP-1), sulfonylureas, dipeptidyl peptidase-4 (DPP-4) inhibitors, colesevelam, thiazolidinediones, meglitinides, or *α*-glucosidase inhibitor, have also been shown with respect to improved glycemic control [11]. However, for morbidly obese patients, bariatric surgery may be the most effective means of treatment [12].

Certain plants have also been used to treat diabetes in traditional medicine. For example,* Nymphaea stellata* extract stimulated glucose-induced insulin secretion and glucose uptake in vitro and insulin response in an in vivo model of type 2 diabetes that was accompanied by increased IRS1 phosphorylation and GLUT4 expression in the liver and muscle [13]. The protective effects of* Astragalus membranaceus* on diabetic nephropathy have also been well established [14, 15]. Both* Astragalus membranaceus* and* Rehmanniae* are used to treat diabetic foot ulcer [16, 17]. In an in vivo model of diet-induced insulin resistance, an* Astragalus membranaceus* polysaccharide (APS) extract improved insulin sensitivity [18]. In a manner similar to that reported for dibenzoylmethane, a structural analog of curcumin, in mice fed a high-fat diet [19], APS reduced hyperglycemia and IR in KKay mice, in part through restoration of PKB phosphorylation and GLUT4 translocation [20]. In 3T3-L1 adipocytes, APS reduced adiponectin and interleukin-6 (IL-6) secretion [21]. Furthermore,* Rehmannia* increased glucose uptake by insulin resistant HepG2 cells [22] and improved insulin sensitivity in an in vivo model of T2DM [23].

Given the effects of APS and* Rehmannia* individually on IR, this study analyzed the effects of another traditional Chinese medicine, Qizhi Jiangtang Jiaonang (QJJ), which is mainly composed of* Astragalus* and* Rehmannia*. HepG2 cells were treated with palmitic acid to induce IR followed by various doses of QJJ after which cell viability, reactive oxygen species (ROS) levels, inflammatory cytokine secretion, and expression of genes in the insulin signaling pathway were analyzed. The effects of QJJ were compared with that of another traditional Chinese medicine, Shenqi Jiangtang Keli (SJK), which is an effective therapy for diabetes [24]. These studies may highlight the efficacy of a traditional Chinese medicine for the treatment of IR.

## 2. Methods

### 2.1. Materials

QJJ, which was comprised of* Astragalus mongholicus*, radices* Rehmanniae*, rhizoma Polygonati, and leech, was obtained from Yizheng Pharmaceutical Group Co., Ltd. (Jilin, China, Traditional Chinese medicine approval number Z10950075; batch number 121011). SJK, which contains panaxoside (cauline leaf),* Schisandra chinensis*, Chinese yam, radices* Rehmanniae*,* Ophiopogon japonicus*,* Astragalus mongholicus*, fructus rubi,* Poria cocos*, radices trichosanthis, rhizoma alismatis, and* Mespilus germanica*, was purchased from Houpu Pharmaceutical Group Co., Ltd. (Lunan, China, Traditional Chinese medicine approval number Z10950075; batch number 1206526).

### 2.2. HepG2 Cultures and Establishment of an IR Model

Human liver cancer HepG2 cells were provided by the Department of Pathogen Biology at Jilin University. HepG2 cells were maintained in RPMI-1640 containing 10% fetal bovine serum (FBS, HyClone, Logan, UT, USA) at 37°C in an environment with 95% O_2_ and 5% CO_2_. When the cell confluence reached approximately 100%, the cells were digested with 0.25% trypsin at 37°C for passaging once every 2 days. For the following experiments, the cell density was adjusted to 1 × 10^5^ cells/mL, and the cells were maintained in medium containing 2% FBS for synchronization.

The IR model was established as previously described [25]. Briefly, HepG2 cells in logarithmic growth phase were seeded onto 96-well plates (100 *μ*L/well) and maintained in RPMI-1640 containing 2% FBS for 24 h after which they were treated with 400 *μ*M palmitic acid for 24 h to induce IR. The palmitic acid solution was prepared as follows: palmitic acid was mixed with 0.1 M NaOH, followed by incubation at 70°C to prepare a 0.1 M sodium palmitate solution, which was then mixed with 5% bovine serum albumin (Calbiochem, Billerica, MA, USA) at a ratio of 1 : 9, and incubated at 37°C for 1 h. The resultant mixture, 10 mM sodium palmitate solution, was filter-sterilized and stored at 4°C until further use.

### 2.3. Analysis of Maximum Nontoxic Concentration of QJJ (*T*_0_)

HepG2 cells in logarithmic growth phase were seeded into 96-well plates at a density of 1 × 10^5^ cells/mL (100 *μ*L/well). Various concentrations of QJJ (0, 5, 10, 20, 40, 80, 160, and 320 *μ*g/mL) were added to each group of 6 wells. After incubation for 24 h, the MTT assay (Chemicon International, Temecula, CA, USA) was performed to analyze cell viability, and a survival curve was produced to determine the maximum nontoxic concentration of QJJ. Cells were also treated with various concentrations of SJK to identify the optimal SJK concentration.

### 2.4. Grouping and Treatments

Cells were divided into the following groups: normal control group, IR group, low-dose QJJ group, intermediate-dose QJJ group, high-dose QJJ group, and SJK group. In the normal control group, untreated cells were maintained in RPMI-1640 containing 2% FBS. In the IR group, cells were treated with 400 *μ*M palmitic acid for 24 h [22]. In the QJJ groups, cells were treated with 400 *μ*M palmitic acid for 24 h and then incubated with QJJ at TC_0_, 1/2 TC_0_, and 1/4 TC_0_ in serum-free RPMI-1640 for 24 h. In the SJK group, cells were treated with 400 *μ*M palmitic acid for 24 h and then incubated with SJK at 1/2 TC_0_ in serum-free RPMI-1640 for 24 h. This experiment was performed three times.

### 2.5. Detection of Cell Viability and Glucose Uptake

After the indicated treatments, cell viability was determined using the MTT assay and was calculated as follows: (OD_experiment_/OD_normal  control_) × 100%. An enzyme-linked immunosorbent assay (ELISA) (Blue Gene, Shanghai, China) was employed to detect the amount of glucose consumed by treated HepG2 cells by examining the glucose concentration in the cell supernatant.

### 2.6. Detection of Reactive Oxygen Species (ROS)

After the HepG2 cells were treated as described above for 24 h and washed twice with serum-free RPMI-1640, they were incubated with 10 *μ*M dichloro-dihydro-fluorescein diacetate (DCHF-DA, Beyotime Institute of Biotechnology, Haimen, China) for 20 min in the dark and washed twice with serum-free RPMI-1640. Cells were harvested and subjected to flow cytometry using a SPACE∖SL∖CUBE flow cytometer (Yiming Biotech Co., Ltd., Shanghai, China).

### 2.7. Detection of Cytokine Secretion

The cell culture supernatant of the treated HepG2 cells was collected, followed by centrifugation at 3000 rpm for 10 min. Tumor necrosis factor-*α* (TNF-*α*), interleukin-1*β* (IL-1*β*), and IL-6 levels were determined using the corresponding ELISA kits (RayBiotech, Norcross, GA, USA).

### 2.8. Real-Time PCR Analysis

The total RNA was extracted from treated HepG2 cells using Trizol (Invitrogen, Carlsbad, CA, USA) and reverse-transcribed into cDNA using the PrimeScript RT reagent kit (TaKaRa, Otsu, Shiga, Japan). PI3K-C2-alpha, IRS-1, GLUT4, RAGE, NF-*κ*B, and JNK mRNA levels were detected by real-time PCR using SYBR Premix Ex Taq (TaKaRa) and an ABI 7300 fluorescence quantitative thermal cycler (Applied Biosystems, Foster City, CA, USA). The primers used in this analysis are shown in Table 1 and were synthesized by Shanghai Sangon Co., Ltd. The PCR reaction was incubated at 95°C for 30 sec, followed by 40 cycles of 95°C for 5 sec and 60°C for 30 sec, with a maximum of 40 cycles. Furthermore, the PCR reaction was finally incubated at 95°C for 15 sec and 60°C for 1 min.

### 2.9. Western Blot Analysis

The treated HepG2 cells were lysed with the lysis buffer (20 mM sodium phosphate pH 6.8, 1% Triton X-100, and 1 mM PMSF) on ice for 30 min followed by centrifugation at 12000 rpm for 30 min at 4°C. After the supernatant was collected, the protein concentration was determined using the BCA method (Beyotime Institute of Biotechnology), according to the manufacturer's instructions. The proteins (20 *μ*g) were subsequently separated by electrophoresis and transferred onto a PVDF membrane (Millipore, Billerica, MA, USA), which was blocked with normal goat serum. After the membranes were incubated with polyclonal primary antibody against RAGE, NF-kB, JNK, IRS-1, PI3K p100 (class III), or GLUT4 (1 : 200, Santa Cruz Biotechnology, Santa Cruz, CA, USA) at 4°C overnight, they were incubated with secondary antibodies (Santa Cruz Biotechnology, Santa Cruz, CA, USA) at 37°C for 1 h and visualized with an ECL kit (Gene Tech, Shanghai, China). Analysis of *β*-actin levels (1 : 1000, Santa Cruz) served as a control.

### 2.10. Statistical Analysis

All the data were analyzed using SPSS 18.0 statistics software (SPSS Inc., Chicago, IL, USA). Data were expressed as mean ± standard deviation (SD) for a given concentration or group. The HepG2 cell survival rates at the various doses were compared with that of untreated cells using the paired *t*-test. For the other data, differences between two groups were compared using two-sample *t*-tests. All the statistical assessments were two-tailed, and *P* values <0.05 were considered significant. 

## 3. Results

### 3.1. Effects of QJJ on HepG2 Cell Survival

**Figure 1:**
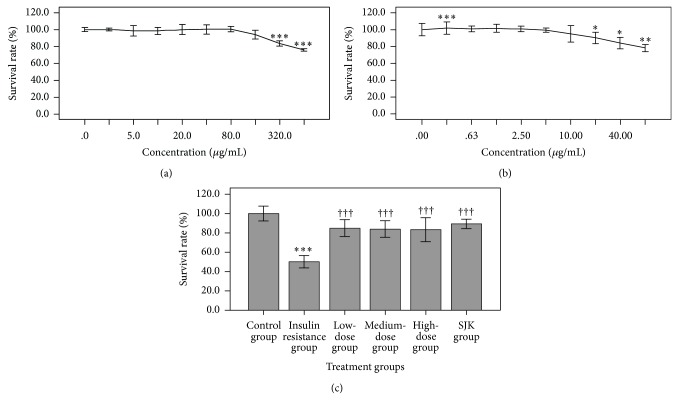
Effects of QJJ and SJK on HepG2 cell survival. HepG2 cells were treated with the indicated concentration of (a) QJJ or (b) SJK. The survival rate was represented as mean ± SD (*n* = 6 per concentration). ^∗^ *P* < 0.05, ^∗^ *P* < 0.01, and ^∗∗∗^ *P* < 0.001, significantly different as compared with the untreated control. (c) Survival rate of HepG2 cells in the untreated control group, IR group, low-dose QJJ (20 *μ*g/mL), intermediate-dose QJJ (40 *μ*g/mL), high-dose QJJ (80 *μ*g/mL), and SJK group (2.5 *μ*g/mL SJK). The survival rate was represented as mean ± SD (*n* = 6 per group, except *n* = 4 for the control group). ^∗∗∗^ *P* < 0.001, significantly different as compared with the control group; ^†††^ *P* < 0.001, significantly different as compared with the IR group.

As shown in Figure 1(a), the HepG2 cell survival rate significantly decreased upon treatment with 320 and 640 *μ*g/mL QJJ as compared to untreated cells (both *P* < 0.001). In addition, the survival rates of HepG2 cells treated with 20, 40, and 80 *μ*g/mL SJK were significantly decreased compared to that of the untreated control group (all *P* < 0.05, Figure 1(b)). Based on these results, HepG2 cells were treated with 20, 40, and 80 *μ*g/mL QJJ as low-, intermediate-, and high-dose treatments. The SJK group was treated with 2.5 *μ*g/mL SJK.

After establishment of the IR model in HepG2 cells, the effects of QJJ on cell survival were next determined. As shown in Figure 1(c), the survival rate for HepG2 cells in the IR group was significantly lower than that of the control group (*P* < 0.001). However, treatment with QJJ at all doses analyzed restored HepG2 cell survival as compared with the IR group (all *P* < 0.001).

### 3.2. Effects of QJJ on HepG2 Cell Glucose Uptake and ROS Levels

The influence of QJJ on glucose uptake by HepG2 cells was next determined after establishment of an IR model. As shown in Table 2, glucose uptake was significantly reduced in HepG2 cells after palmitic acid treatment in the IR group as compared to the normal control group (*P* < 0.05). As compared with HepG2 cells in the IR group, QJJ at all doses analyzed significantly increased glucose consumption (all *P* < 0.05); similar effects were observed in the SJK group (*P* < 0.05). However, the glucose uptake by the QJJ-treated cells was compared to that of the SJK group (Table 2).

**Figure 2:**
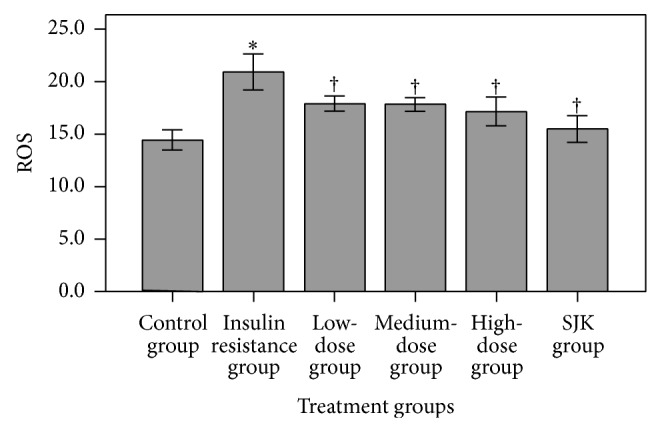
Effects of QJJ on HepG2 cell ROS levels. ROS values for the untreated control group, IR group, low-dose QJJ (20 *μ*g/mL), intermediate-dose QJJ (40 *μ*g/mL), high-dose QJJ (80 *μ*g/mL), and SJK group (2.5 *μ*g/mL SJK) were determined. The ROS value was represented as mean ± SD by group (*n* = 3 per group). ^∗^ *P* < 0.05, significantly different as compared with the control group; ^†^ *P* < 0.05, significantly different as compared with the IR group.

Analysis of ROS levels in the HepG2 cells revealed increased levels in the IR group as compared to the untreated control cells (*P* < 0.05, Figure 2). Moreover, treatment with all the QJJ doses as well as SJK significantly reduced the mean ROS levels as compared with the IR group (all *P* < 0.05).

### 3.3. QJJ Reduces Inflammatory Cytokine Levels in an IR Model in HepG2 Cells

**Figure 3:**
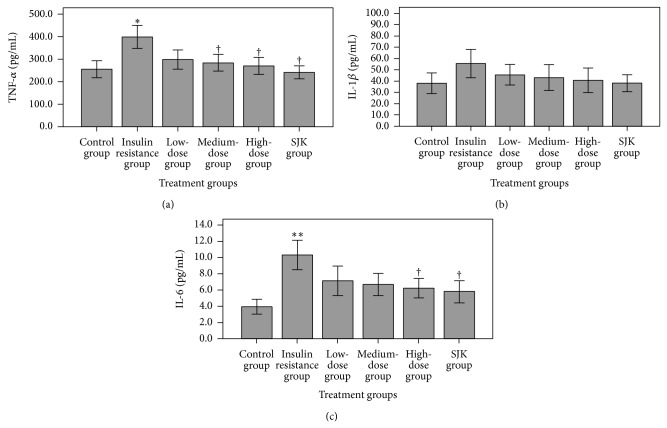
QJJ reduces inflammatory cytokine levels in an IR model in HepG2 cells. Protein levels of TNF-*α* (a), IL-1*β* (b), and IL-6 (c) by HepG2 cells of the untreated control group, IR group, low-dose QJJ (20 *μ*g/mL), intermediate-dose QJJ (40 *μ*g/mL), high-dose QJJ (80 *μ*g/mL), and SJK group (2.5 *μ*g/mL SJK) were determined. TNF-*α*, IL-1*β*, and IL-6 levels were represented as mean ± SD (*n* = 3 per group). ^∗^ *P* < 0.05, ^∗∗^ *P* < 0.01, significantly different as compared with the control group; ^†^ *P* < 0.05, significantly different as compared with the IR group.

The effects of QJJ on inflammatory cytokine secretion were next assessed in the in vitro IR model. TNF-*α* and IL-6 levels were significantly higher in the IR group than the control group (both *P* < 0.05, Figures 3(a) and 3(c), resp.). However, their levels were significantly reduced with high-dose QJJ as well as SJK as compared to the IR group (all *P* < 0.05). However, no changes in IL-1*β* levels were observed among the treatment groups (all *P* > 0.05, Figure 3(b)).

### 3.4. QJJ Ameliorates the Changes in Gene Expression Induced by IR

**Figure 4:**
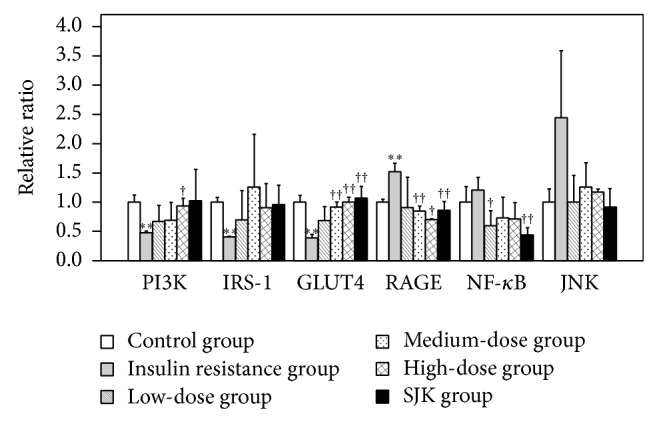
QJJ ameliorates the changes in gene expression induced by IR. The relative mRNA expression of PI3K, IRS-1, GLUT4, RAGE, NF-*κ*B, and JNK by HepG2 cells of the control group, IR group, low-dose QJJ (20 *μ*g/mL), intermediate-dose QJJ (40 *μ*g/mL), high-dose QJJ (80 *μ*g/mL), and SJK group (2.5 *μ*g/mL SJK) was determined. The relative level was represented as mean ± SD (*n* = 3 per group). ^∗^ *P* < 0.05, ^∗∗^ *P* < 0.01, significantly different as compared with the control group; ^†^ *P* < 0.05, ^††^ *P* < 0.01, significantly different as compared with the IR group.

We next analyzed the effects of QJJ on the expression of genes known to be altered with IR, including PIK3, IRS-1, GLUT4, RAGE, NF-KB, and JNK. As shown in Figure 4, the relative mRNA expression of PI3K, IRS-1, and GLUT4 by HepG2 cells in the IR group was significantly lower than that observed in the untreated control group (all *P* < 0.05); RAGE mRNA expression was significantly higher in the IR group (*P* < 0.05). In addition, as compared to the IR group, the high-dose QJJ group had significantly higher relative PI3K, GLUT mRNA expression and significantly reduced RAGE mRNA expression (all *P* < 0.05). Furthermore, the low-dose QJJ group had significantly lower relative mRNA expression of NF-*κ*B than the IR group (*P* < 0.05). There was no significant difference among the groups in JNK mRNA level (Figure 4).

**Figure 5:**
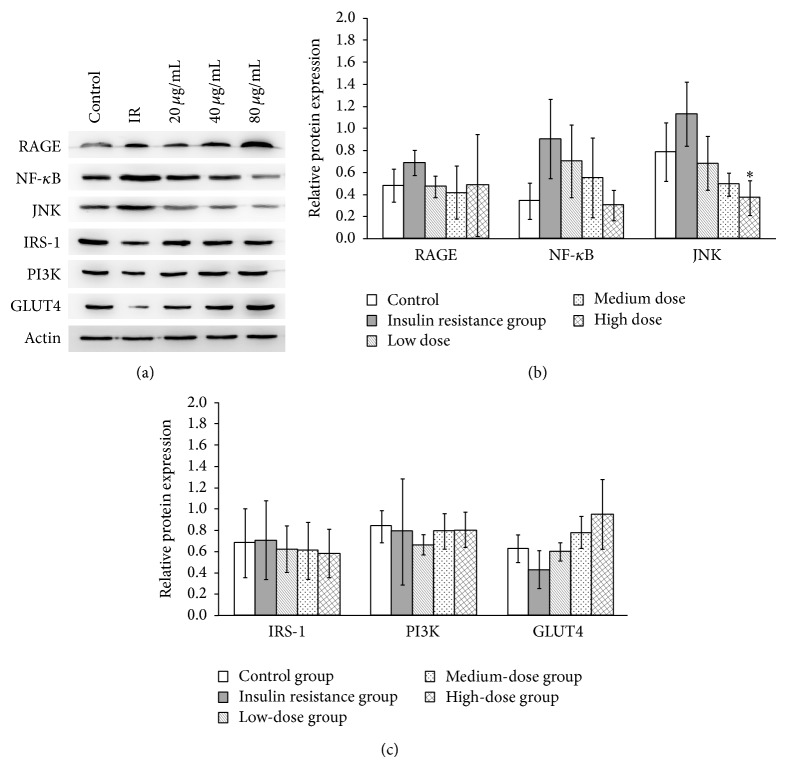
Effects of QJJ on RAGE, NF-*κ*B, JNK, IRS-1, PI3K, and GLUT4 protein expression. Protein expression was determined in the HepG2 cells of the control group, IR group, low-dose QJJ (20 *μ*g/mL), intermediate-dose QJJ (40 *μ*g/mL), high-dose QJJ (80 *μ*g/mL), and SJK group (2.5 *μ*g/mL SJK) by Western blot analysis. (a) A representative gel image is shown. The levels of (b) RAGE, NF-*κ*B, and JNK as well as (c) IRS-1, PI3K, and GLUT4 protein expression were quantified, and the data were presented as mean ± SD (*n* = 3 for each group). ^∗^ *P* < 0.05, significantly different as compared with the IR group.

The effects of QJJ on PIK3, IRS-1, GLUT4, RAGE, NF- *κ*B, and JNK protein levels were also determined using Western blot analysis (Figure 5(a)). As compared with the normal control group, RAGE, NF-*κ*B, and JNK protein levels were increased in the IR group (Figure 5(b)), and GLUT4 was reduced although the difference is not significant (Figure 5(c)). QJJ significantly downregulated the protein expression of JNK protein expression as compared to the IR group.

## 4. Discussion

The effects of QJJ on cell viability, insulin signaling, ROS production, and inflammatory cytokine secretion were analyzed in insulin resistant HepG2 cells. QJJ not only increased cell survival but also reduced ROS levels in palmitic acid-treated cells. In addition, reduced secretion of TNF-*α* and IL-6 by insulin resistant HepG2 cells was observed. Finally, QJJ ameliorated the altered expression of PI3K, GLUT4, and RAGE observed with IR.

In the present study, HepG2 cells were treated with 400 *μ*M palmitic acid for 24 h to establish IR, which was confirmed by reduced glucose consumption from 3.03 ± 0.31 mmol/L to 2.18 ± 0.26 mmol/L and decreased cell viability. These data suggest that palmitic acid may induce the apoptosis in HepG2 cells and reduce glucose uptake by these cells, indicating that the IR model was successfully established.

In fibroblasts isolated from diabetic patients, both* Astragalus* and* Rehmanniae* increased cell viability [17], which is consistent with the increased viability with QJJ observed in the present study (from 50.26% to 84%). In addition, we observed increased glucose consumption by HepG2 cells with QJJ treatment, which may be due in part through increased uptake via GLUT4. Although glycemia- and hormone-induced (insulin, glucagon, glucocorticoids, growth hormone, and epinephrine) glucose production is the best characterized mechanism of glucose homeostasis by the liver [26], roles for hepatic glucose uptake have also been reported. For example, overexpression of glucokinase (GK), which promotes pancreatic *β* cell secretion of insulin and hepatic glucose uptake, in the liver, results in a reduction in plasma glucose levels as well as improved glucose tolerance [27]. However, long-term activation of GK by a small molecule activator, GKA71, did not alter glycogen or triglyceride levels in the livers of mice fed a high-fat diet [28]. In addition, the liver is responsible for 70–80% of insulin clearance through insulin-degrading enzyme, a process that is compromised in nutrient restriction [29].

The effects of QJJ on HepG2 cell viability and glucose uptake suggest that it can attenuate the apoptosis and necrosis of HepG2 cells and increase the glucose uptake, exerting protective effect on IR. This is in contrast to a study by Ma et al. [30] in which* Astragalus membranaceus *was ineffective at increasing insulin sensitivity of dexamethasone-induced insulin resistant HepG2 cells. The inconsistencies may be due to the in vitro IR model employed as APS improved glucose uptake by C2C12 skeletal muscle myotubes after induction of palmitate-induced IR [31], and* Rehmannia* increased glucose uptake by insulin resistant HepG2 cells [22]. Alternatively, other components of QJJ, including* Rehmannia*, may be mediating the effects. Therefore, further analysis of the in vivo effects of QJJ on IR is required to confirm our study.

The pathogenesis of IR is mediated in part through altered IRS-1/PI3K/GLUT signaling. In C2C12 skeletal muscle myotubes treated with palmitate to induce IR, APS increased Ser473 phosphorylation of Akt [31]. Similarly, in the present study, QJJ suppressed the impaired insulin signaling induced by palmitate by increasing PI3K and GLUT4 expression, suggesting that it could positively induce insulin signaling in a manner similar to that reported for Silibinin [32]. These results are consistent with a previous study reporting increased GLUT4 mRNA expression with an* Astragalus* and* Rehmanniae* herb formula [17].

In addition to IRS-1/PI3K/GLUT signaling, the pathogenesis of IR is also related to inflammation and oxidative stress, and some IR treatments, including peroxisome proliferator-activated receptor *δ* agonist, function through an anti-inflammatory mechanism [33]. Specifically, hyperglycemia may facilitate the synthesis of advanced glycation end products (AGEs) that induce oxidative stress via RAGE, activating NF-*κ*B that initiates and regulates inflammation by synthesizing proinflammatory cytokines, including TNF-*α*, IL-1*β*, and IL-6 [34]. These proinflammatory cytokines may, in turn, further activate NF-*κ*B as well as inhibiting the insulin-induced tyrosine phosphorylation of IRS and PI3K in IR. In the present study, QJJ decreased the expression of RAGE as well as the secretion of TNF-*α* and IL-6; ROS levels were also markedly reduced by QJJ, suggesting that it can inhibit inflammation- and oxidative stress-related signal transduction. This is consistent with a previous report of APS function in preventing diabetes in NOD mice [35] as well as another study showing the anti-inflammatory effects of* Rehmannia* [36]. Furthermore, increased JNK levels were observed in the IR group, which is consistent with that reported by Gao et al. [37], and these levels were decreased by both QJJ and SJK. However, the specific mechanisms underlying the therapeutic effects of QJJ remain unknown and require further investigation.

The present study is limited by its in vitro nature; therefore, in vivo analyses are required to confirm the effects of QJJ. Furthermore, although QJJ increased the survival of insulin resistant HepG2 cells, the mechanism underlying this effect was not determined. Further studies will assess the effects of QJJ on cell apoptosis in IR. Finally, QJJ is comprised of a mixture of compounds that are primarily derived from* Astragalus* and* Rehmannia*. Additional studies will isolate the compound(s) responsible for attenuating the effects of IR.

## 5. Conclusions

QJJ can improve IR in HepG2 cells, which may be mediated through the IRS-1/PI3K/GLUT4 signaling pathway as well as regulation of NF-*κ*B-mediated inflammation and oxidative stress. Further in vivo analyses are required to confirm these findings as well as uncovering the mechanism through which QJJ improves IR.

## Figures and Tables

**Table 1:** Primers for real-time PCR analysis.

Genes	Forward (5′-3′)	Reverse (5′-3′)
*β*-actin	CTGGAACGGTGAAGGTGACA	AAGGGACTTCCTGTAACAATGCA
PI3K	TGGGACCAGTAGTTTGCCAACTGG	AGCCTGGGTTTGTGCGGTGATT
IRS1	TTTAAGCGCCTATGCCAGC	TTAGAGTCTGGGTACCCATGAG
Glut4	ACAGTCTTCACCTTGGTCTC	GCAGCTGAGATCTGGTCAAA
RAGE	GGCTGGTGTTCCCAATAAGG	TCACAGGTCAGGGTTACGGTTC
NF-*κ*B	TGCTGTGCGGCTCTGCTTCC	AGGCTCGGGTCTGCGTAGGG
JNK1	TGGACTTGGAGGAGAGAACCAAGA	AGCCATTGATCACTGCTGCACCT

**Table 2:** Effects of QJJ on glucose uptake by HepG2 cells.

Groups	Dose (*μ*g/mL)	Glucose consumption (mmol/L)
Normal control	—	3.03 ± 0.31^∗∗^
Insulin resistance	—	2.18 ± 0.26
QJJ	20	2.94 ± 0.32^∗∗^
	40	2.97 ± 0.38^∗∗^
	80	3.01 ± 0.41^∗∗^
SJK	2.5	3.21 ± 0.41^∗∗^

^∗∗^ *P* < 0.01 versus the insulin resistance group.
